# Draft Genome Sequence of Acholeplasma laidlawii Isolated from the Conjunctiva of a Heifer with Infectious Bovine Keratoconjunctivitis

**DOI:** 10.1128/MRA.01345-20

**Published:** 2021-01-28

**Authors:** Gloria Gioia, Maria F. Addis, Laura B. Goodman, Patrick K. Mitchell, Belinda Thompson, Erin Goodrich, Paolo Moroni

**Affiliations:** a Animal Health Diagnostic Center, Cornell University, Ithaca, New York, USA; b Università degli Studi di Milano, Dipartimento di Medicina Veterinaria, Lodi, Lombardy, Italy; University of Arizona

## Abstract

Acholeplasma laidlawii can be isolated from cattle environments and different body sites of bovines. It is still under evaluation if *A. laidlawii* acts as a primary pathogen. Here, we present the whole-genome sequence of *A. laidlawii* isolated from the conjunctiva of a heifer with infectious bovine keratoconjunctivitis.

## ANNOUNCEMENT

Acholeplasmas are cell wall-less bacteria belonging to the class *Mollicutes*, order *Acholeplasmatales*, and classified in the family *Acholeplasmataceae*. *Acholeplasma* spp. are described as saprophytes found in soil, compost, and wastewater or commensals distributed in vertebrates, insects, and plants (1).

Within the *Acholeplasma* genus, Acholeplasma laidlawii is the best-studied species. *A. laidlawii* is often found in dairy and beef cattle environments. It has been isolated from various bovine body sites, including mastitic milk, bulk tank milk, aborted fetuses, semen, preputial samples, nasal secretions, pneumonia, and healthy lungs (2, 3). One trial performed by Pugh et al. in 1976 (4) demonstrated the ability of *A. laidlawii* to establish clinical signs of infectious bovine keratoconjunctivitis in calves after experimental induction. Here, we announce the draft genome sequence of *A. laidlawii* strain QMP CG1-1743, isolated from a conjunctival swab sample from a 6- to 9-month-old heifer affected by infectious bovine keratoconjunctivitis and showing watery eyes and corneal opacities. The heifer tested was from an organic herd; it was not treated with any antibiotics but was administered plasma eye drops as treatment. The initial isolation of strain QMP CG1-1743 was carried out using standard procedures for mycoplasma culturing, which include streaking on modified Hayflick agar medium and incubation for up to 7 days at 37°C with CO_2_ enrichment. Colonies showing typical mycoplasma morphology were submitted for molecular confirmation, and further identification of isolates to the species level was performed by a PCR amplification of the 16S-23S internal transcribed spacer (ITS) followed by Sanger sequencing of the amplicons (5).

The isolate was then subcultured in modified Hayflick medium, incubated for 3 days at 37°C with CO_2_, and pelleted by centrifugation at 13,000 × *g* for 10 min. DNA was directly extracted from the pellet using a MagMAX core nucleic acid purification kit (Applied Biosystems, Foster City, CA). DNA concentration was measured with a Qubit 3.0 fluorometer (Life Technologies, MD). Library preparation was performed using an Illumina Nextera XT prep kit. The library was sequenced on an Illumina MiSeq device using 2 × 250-bp reads, and read quality was assessed using FastQC (https://www.bioinformatics.babraham.ac.uk/projects/fastqc/) (6). A total of 406,301 paired reads were generated with an average Phred score of 37.35 for forward reads and 37.12 for reverse reads. Raw reads were assembled using SKESA v. 2.3.0 (7), assembly quality was checked using QUAST v. 5.0.2 (8), and reads were mapped back to the assembly to identify coverage depth with BBMap v. 38.58 (http://sourceforge.net/projects/bbmap/). Total assembly length was 1,329,497 bp with 55 contigs, an *N*_50_ value of 75,018 bp, and 31.74% GC content. The average read depth was 108×. BUSCO v. 4.1.4 was used to assess sequence completeness (9). Complete copies of all 151 genes in the mollicutes_odb10 data set (created 6 March 2020) were recovered, with 149/151 as single copies and 2/151 duplicated. Average nucleotide identity (ANI) was computed against all *Acholeplasma* genomes available from GenBank (accessed 7 July 2020) using fastANI v. 1.3. The highest ANI was 93.8% with *A. laidlawii* strain MDBK/IPV (GenBank assembly number GCA_001730135.1). The ANIs compared to three assemblies of the *A. laidlawii* type strain (GCA_000018785.1, GCA_900476025.1, and GCA_003385765.1) were 93.2 to 93.3%, indicating that this isolate is divergent from previously sequenced *A. laidlawii* isolates and might represent a distinct lineage.

### Data availability.

This whole-genome shotgun project has been deposited at DDBJ/ENA/GenBank under the accession number JADNRS000000000. The version described in this paper is version JADNRS010000000. The reads are available through the NCBI Sequence Read Archive under accession number SRR12739111.
